# Cardiorespiratory monitoring of red blood cell transfusions in preterm infants

**DOI:** 10.1007/s00431-021-04218-5

**Published:** 2021-08-09

**Authors:** Jarinda A. Poppe, Tanja van Essen, Willem van Weteringen, Sten P. Willemsen, Irwin K. M. Reiss, Sinno H. P. Simons, Rogier C. J. de Jonge

**Affiliations:** 1 Department of Pediatrics, Division of Neonatology, Erasmus MC Sophia Children’s Hospital, University Medical Center Rotterdam, Rotterdam, Netherlands; 2 Department of Pediatric Surgery, Erasmus MC Sophia Children’s Hospital, University Medical Center Rotterdam, Rotterdam, Netherlands; 3 Department of Biostatistics, Erasmus MC, Erasmus University Medical Center, Rotterdam, Netherlands; 4 Pediatric Intensive Care Unit, Department of Pediatrics and Pediatric Surgery, Erasmus MC Sophia Children’s Hospital, University Medical Center Rotterdam, Rotterdam, Netherlands

**Keywords:** Red blood cell transfusion, Vital signs, Preterm infants, Hypoxia, Clinical decision-making

## Abstract

Clinical improvement after red blood cell (RBC) transfusions in preterm infants remains debated. This study aims to investigate the effect of RBC transfusion on the occurrence of desaturations and hypoxia, and other cardiorespiratory outcomes in preterm infants. In this longitudinal observational study, prospectively stored cardiorespiratory parameters of preterm infants who received at least one RBC transfusion between July 2016 and June 2017 were retrospectively analyzed. Sixty infants with 112 RBC transfusions, median GA of 26.7 weeks, were included. The number of desaturations and area < 80% SpO_2_ limit, as a measure of the hypoxic burden, were calculated in 24 h before and after RBC transfusion. A mixed effects model was used to account for repeated measurements. Overall, the mean (SE) number of desaturations per hour decreased from 3.28 (0.55) to 2.25 (0.38; *p* < 0.001), and area < 80% SpO_2_ limit decreased from 0.14 (0.04) to 0.08 (0.02) %/s (*p* = 0.02). These outcomes were stratified for the number of desaturations in 24 h prior to RBC transfusion. The largest effect was observed in the group with the highest mean number of desaturations (≥ 6) prior to RBC transfusion, with a decrease from 7.50 (0.66) to 4.26 (0.38) (*p* < 0.001) in the number of desaturations and 0.46 (0.13) to 0.20 (0.06) in the area < 80% SpO_2_. Perfusion index increased significantly after RBC transfusion (*p* < 0.001). No other significant effects of RBC transfusion on cardiorespiratory data were observed.

*Conclusions*: RBC transfusions in preterm newborns could help decrease the incidence of desaturations and the area < 80% SpO_2_ as a measure of the hypoxic burden. The higher the number of desaturations prior to the RBC transfusion, the larger the effect observed.

**Table:** 

**What is Known:** *•Red blood cell transfusions potentially prevent hypoxia in anemic preterm infants by increasing the circulatory hemoglobin concentration and improving tissue oxygenation.* *•There is not a predefined hemoglobin concentration cut-off for the occurrence of symptomatic anemia in preterm infants.*	
**What is New:** *•Oxygen desaturations and hypoxia in anemic preterm infants can be improved by RBC transfusions, especially if more desaturations have occurred before transfusion.* *•Cardiorespiratory monitor data may help identify infants who will benefit most from red blood cell transfusions.*

**Supplementary information:**

The online version contains supplementary material available at 10.1007/s00431-021-04218-5.

## Introduction

Preterm infants often become anemic in the first weeks after birth and may require red blood cell (RBC) transfusion. A study found that the incidence of RBC transfusion is inversely correlated to birth weight with 25% to 82% of very low birth weight infants receiving at least one RBC transfusion [1]. Anemia in preterm infants is associated with an increased occurrence of apnea. A possible cause is decreased oxygen transport capacity to the central nervous system followed by lower efferent output of the respiratory center, thus resulting in central apneas [2]. Anemic patients receive RBC transfusions to prevent hypoxia by increasing circulatory hemoglobin (Hb) and improving tissue oxygenation.

Despite extensive research, the optimal RBC transfusion policy for preterm infants remains unclear [3, 4]. Although some studies found transfusions to be effective in preventing apneas and reducing intermittent hypoxia on the short term, several other studies reported minimal or no effect [5–10]. Additionally, retrospective studies found that RBC transfusions in preterm infants were associated with an increased incidence of intra-hospital mortality, intraventricular hemorrhage, and necrotizing enterocolitis [11–16]. A systematic review concluded, however, that no difference in mortality and severe morbidity at hospital discharge was found in clinical trials comparing liberal to restrictive transfusion strategy [17]. Two recent RCTs reported in addition no difference between liberal and restrictive transfusion strategies in death and/or neurodevelopmental impairment in the long term [18, 19].

Identifying those individual patients who will benefit from RBC transfusion remains difficult. Various potential indicators, such as peripheral fractional oxygen extraction, cerebral regional saturation, lactic acid, and vascular endothelial growth factor, have been described [20–23]. According to an international survey, the degree of oxygen requirement and the need for respiratory support are important determinants for the need for transfusion [24]. Although patients are monitored continuously during intensive care admission, only a snapshot of this information is presented to clinicians at the bedside. Visualizing and analyzing these trend data could improve the assessment of medical interventions.

We hypothesized that RBC transfusion decrease the incidence of desaturations and hypoxia in preterm infants and explored this hypothesis by analyzing cardiorespiratory monitor data for different gestational ages (GA), types of respiratory support, and hematocrit (Ht) values before transfusion. Additionally, we evaluated the effect of RBC transfusion on fraction of inspired oxygen (FiO_2_), heart rate, respiratory rate, perfusion index, and blood pressure.

## Materials and methods

### Study design and population

In this longitudinal observational study, prospectively stored physiological data were analyzed in retrospect. Preterm infants who received at least one RBC transfusion between July 2016 and June 2017 at the neonatal intensive care unit (NICU) of the Erasmus MC Sophia Children’s Hospital were eligible for inclusion. We excluded data when patients had received an exchange transfusion for hyperbilirubinemia, had been diagnosed with hemolytic anemia, or had died within 24 h after birth. Additionally, individual transfusions administered during surgery, within 24 h after birth, or with a follow-up duration of less than 24 h were excluded. The local medical ethics review board waived approval for this study pursuant to the Dutch Medical Research Involving Human Subjects Act (MEC-2018–1106).

### Data acquisition

Baseline characteristics and pre-transfusion laboratory results were collected from the electronic medical records (HiX version 6.1, Chipsoft, Amsterdam, Netherlands). Small for gestational age was calculated according to Fenton and Kim [25]. The administered volume of blood, the ventilation mode, caffeine therapy, and FiO_2_ were collected from the electronic patient data management system (PICIS, Wakefield, MA). Continuously logged physiological data (1 Hz), automatically collected from bedside monitors (Dräger Infinity® M540, Dräger, Lübeck, Germany) from 72 h before until 72 h after RBC transfusion, included peripheral oxygen saturation (SpO_2_), heart rate, respiratory rate, arterial blood pressure, and perfusion index.  

### Transfusion protocol

A RBC transfusion of 15 ml/kg was administered during 4 h following the local protocol. From 24 h after birth, transfusion was indicated in patients without clinical symptoms and Ht < 18%. Patients with clinical symptoms were transfused at Ht ≤ 30% in case of mean airway pressure (MAP) ≥ 8 cm H_2_O and/or FiO_2_ ≥ 0.4; at Ht ≤ 25% for MAP < 8 cm H_2_O and/or FiO_2_ < 0.4, NIPPV, or CPAP ≥ 6 cm H_2_O; and at Ht ≤ 20%, for CPAP < 6 cm H_2_O. Clinical symptoms were defined as recurrent apneas or bradycardia, increased oxygen need, increased heart rate or respiratory rate during 24 h, and insufficient growth.

### Outcome measures

**Fig. 1:**
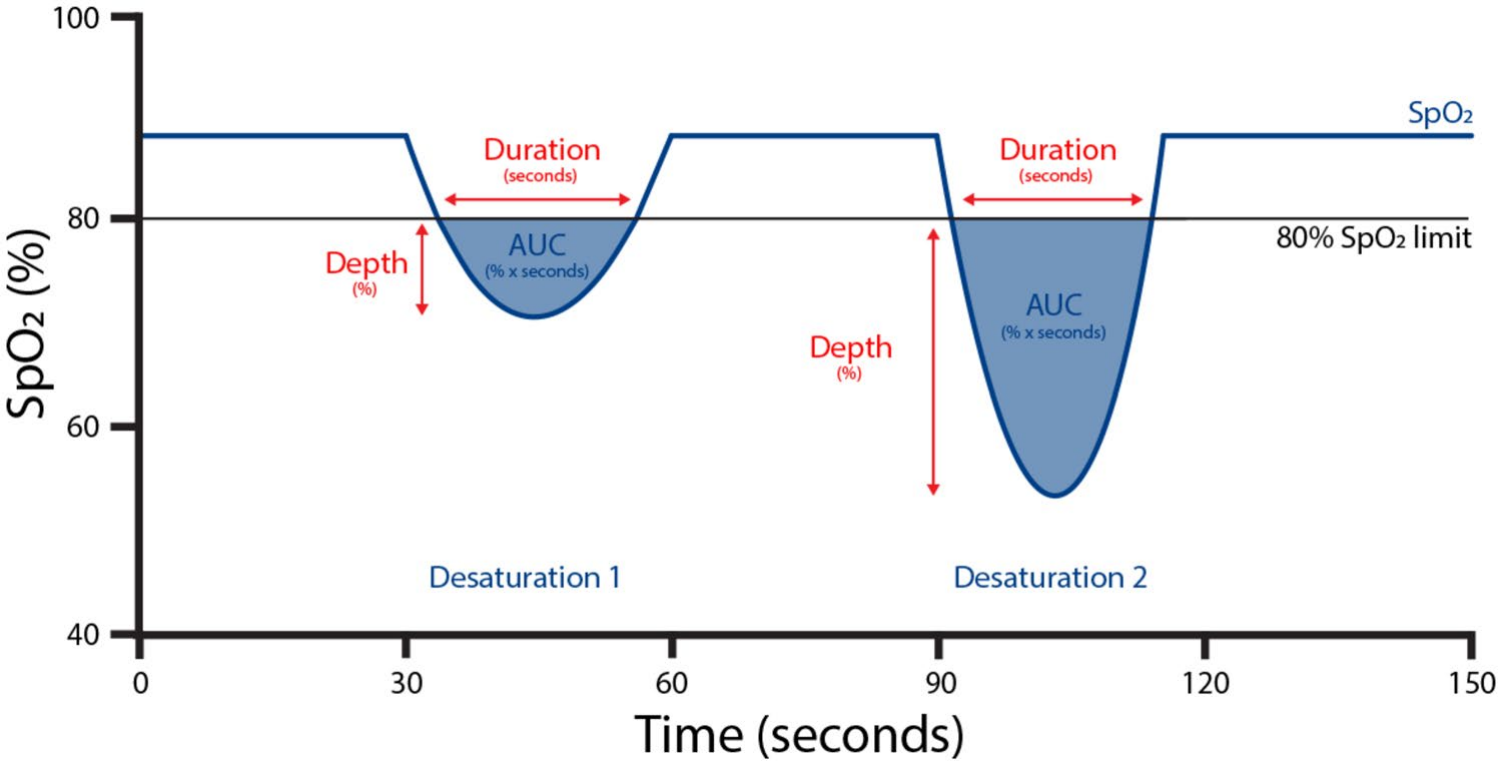
Schematic figure of the number of desaturations and area under the curve (AUC) below a saturation limit of 80%. A desaturation was defined as a saturation level below 80% for a second or longer. The area under the 80% SpO_2_ limit (AUC) was calculated by multiplying the duration (second) by the depth (%) of a desaturation

The primary outcomes are the number of desaturations below an 80% SpO_2_ limit and the area under the 80% SpO_2_ limit as a measure of the hypoxic burden. The limit of 80% was based on the previous data by Poets et al. [26]. The area under the 80% SpO_2_ limit was calculated by multiplying the difference between the SpO_2_ limit and the measured SpO_2_ by the time spent below the SpO_2_ limit, expressed as percent per second (Fig. 1). Secondary outcomes are the FiO_2_, SpO_2_, heart rate, respiratory rate, arterial blood pressure, and perfusion index.

Different subgroups were defined. One is based on the mean number of desaturations in the 24 h before transfusion start, classified as desaturations ≤ 1, desaturations 1–3, desaturations 3–6, and desaturations ≥ 6. Other subgroups were based on GA classified as GA < 26 weeks, GA 26–28 weeks, and GA ≥ 28 weeks, respiratory support at start of transfusion, invasive versus non-invasive, and Ht values prior to transfusion, classified as Ht < 25%, Ht 25–30%, and Ht ≥ 30%.

### Data processing

Measurements marked as invalid by the bedside monitors were excluded. The number of desaturations and the area under the 80% SpO_2_ limit were calculated per hour using LabVIEW (version 2018 SP1, National Instruments, Austin, TX, USA). We preprocessed the secondary outcomes by calculating the median per transfusion for every hour. Data were visualized hourly in median [interquartile range (IQR)] per desaturation subgroup for the primary outcomes and for all transfusions for the secondary outcomes. Data that overlapped with data from the analysis period of an earlier transfusion were excluded from the analysis.

### Statistical analysis

Baseline characteristics are expressed as median [IQR] for continuous variables and as number (%) in categorical variables. Mixed effects models were used in the 24 h before and after RBC transfusion to analyze the effect of transfusion on the outcome of interest with adjustment for repeated measurements. A Poisson mixed effects model was used for count data. Log transformation was applied in case of non-normality of a continuous outcome. Results are presented as estimated means over the 24 h period and standard error (SE). The number of desaturations is presented as mean number per hour and the area under the 80% SpO_2_ limit as percentage per second. All models were adjusted for birth weight, GA, postnatal age at time of RBC transfusion, Ht value before RBC transfusion, respiratory support, and additional caffeine loading doses in 24 h prior to RBC transfusion. A two-sided *p* value of < 0.05 was considered statistically significant. Statistical analyses were performed using the computing environment R (v3.4.1, Inc., Boston, MA, USA) [27]. No imputation methods for missing data were applied.

## Results

**Fig. 2:**
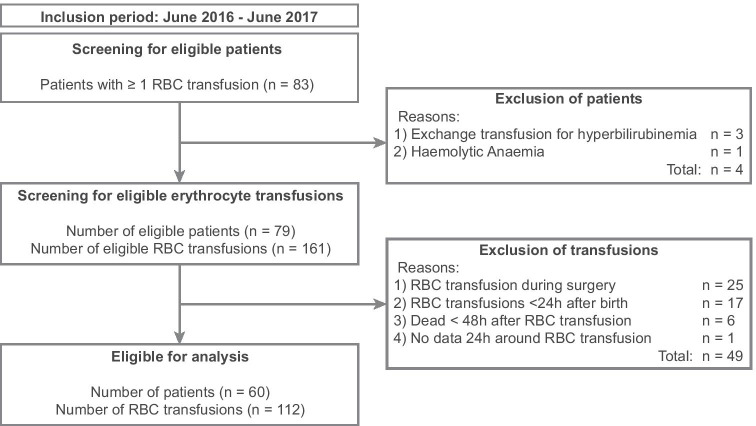
Exclusion of non-eligible preterm infants and red blood cell (RBC) transfusions

Eighty-three preterm infants received at least one RBC transfusion. After exclusion of non-eligible patients and transfusions, data of 60 infants with a total of 112 RBC transfusions were included in the analysis (Fig. 2), of which 47/112 (42%) were first RBC transfusions. The median GA was 26.7 [25.6–29.0] weeks; the median birth weight was 825 [708–959] g (Table 1). During NICU admission, the infants received a median of 2 [1-3] RBC transfusions with a median administered blood volume of 15 [12-18] ml per transfusion. The median postnatal age at RBC transfusion was 19.5 [10.8–29.0] days.

**Table 1:** Demographics and clinical characteristics of the study population

	**Characteristics**	***N***		
Patients (*N* = 60)	Female gender	60	19 (31.1%)	
	Gestational age at birth, weeks	60	26.7 [25.6–29.0]	
	Birth weight, grams	60	825 [708–959]	
	SGA	60	19 (31.7%)	
	Cesarean section	60	36 (60.0%)	
	Singleton	60	46 (76.7%)	
	NEC	60	8 (13.3%)	
	Septic episode during admission	60	35 (59.3%)	
	PDA	60	44 (73.3%)	
	RDS	60	58 (96.7%)	
	BPD	51	31 (60.8%)	
	IVH	60	14 (23.3%)	
	Mortality	60	10 (16.7%)	
	Number of erythrocyte transfusions	60	2 [1–3]	
	Days on invasive mechanical ventilation	60	9 [4–17]	
	Total days of respiratory support	52	72 [42–93]	
Transfusions (*N* = 112)	Number of RBC transfusion			
	1st RBC transfusion	112	47 (42%)	
	2nd RBC transfusion	112	30 (27%)	
	3th RBC transfusion	112	17 (15%)	
	≥ 4th RBC transfusion	112	18 (16%)	
	Postnatal age, days, *at transfusion*	112	19.5 [10.8–29.0]	
	Hemoglobin, mmol/L, *before transfusion*	110	5.9 [5.5–6.8]	
	Hematocrit, %, *before transfusion*	112	27 [25–29]	
	PaO2, kPa, *before transfusion*	103	5.1 [4.2–7.4]	
	PaCO2, kPa, *before transfusion*	109	7.2 [6.4–8.2]	
	According to local transfusion guideline	112	98 (88%)	
	Active bleeding	112	2 (2%)	
	Iron supplementation, *at transfusion*	112	24 (21%)	
	Administered volume of blood, ml	110	15.0 [12.0–18.0]	
	Respiratory support during RBC transfusion	112		
	Invasive respiratory support		74 (66%)	
	Non-invasive respiratory support		38 (34%)	
	Caffeine therapy	112	107 (96%)	
	Loading dose 24 h before transfusion	112	16 (14%)	
	Time before transfusion, *hours*	16	8.0 [5.9–9.3]	

Values are expressed as median [IQR] or number (%); *SGA*, small for gestational age; *NEC*, necrotizing enterocolitis; *PDA*, patent ductus arteriosus; *RDS*, respiratory distress syndrome; *BPD*, bronchopulmonary dysplasia; *IVH*, intraventricular hemorrhage; *RBC* transfusion, red blood cell transfusion

### Number of desaturations and area under the 80% SpO_2_ limit

**Fig. 3:**
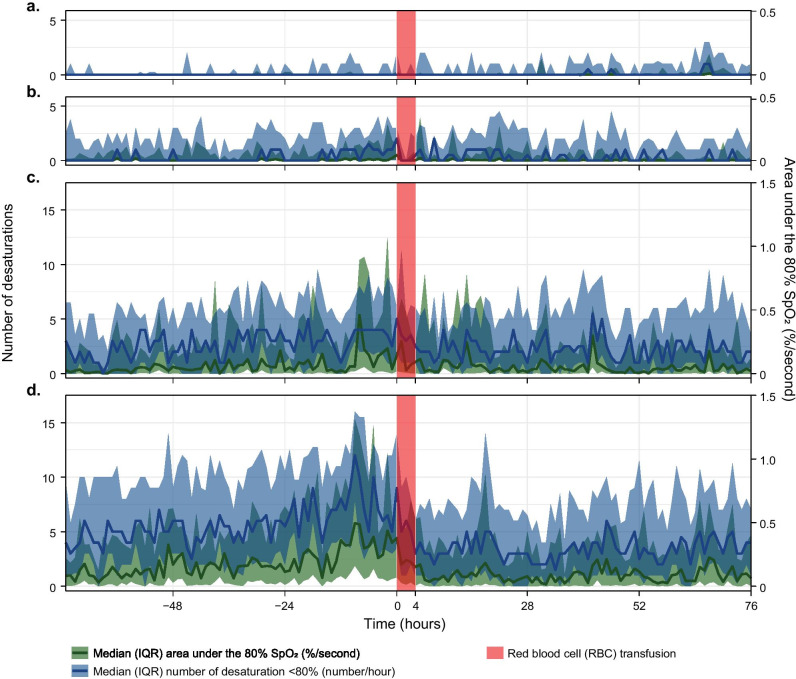
Graphs with data of the red blood cell (RBC) transfusions per desaturation subgroup, from 3 days before until 3 days after transfusion. The number of desaturations per hour on the left *y*-axis and the area under the 80% oxygen saturation on the right *y*-axis are displayed for RBC transfusions with mean number of desaturations in the 24 h prior to transfusion **a** ≤ 1, **b** 1–3, **c** 3–6, and **d** ≥ 6. Data are presented as median [interquartile range (IQR)] because of the skewed distribution

Overall, the number of desaturations per hour in the 24 h before transfusion decreased from mean (SE) 3.28 (0.55) to 2.25 (0.38) in the 24 h after transfusion (*p* < 0.001) (Table 2). The area under the 80% SpO_2_ limit decreased from 0.14 (0.04) to 0.08 (0.02) %/s in all RBC transfusions (*p* = 0.02). The decrease in the number of desaturations and the area under the 80% SpO_2_ limit was most prominently in those with higher mean desaturations (Fig. 3a–d). In contrast to the area under the 80% SpO_2_ limit, the number of desaturations decreased significantly in all GA subgroups and Ht subgroups (Table 2). The number of desaturations decreased significantly in both the invasive and the non-invasive respiratory support groups, whereas the area under the 80% SpO_2_ limit decreased significantly only in the invasive respiratory support group (Supplemental Fig. 1a, b; Table 2).

**Table 2:** Before and after red blood cell transfusion comparisons in the number of desaturations and the area under 80% SpO_2_ limit

**Subgroup**		**Outcome**	**Pre-RBC transfusion**	**Post-RBC transfusion**	*p *value
**All RBC transfusions**	*N = 112*	Area < 80% SpO_2_ limit	0.14 (0.04)	0.08 (0.02)	0.02
		No of desaturations	3.28 (0.55)	2.25 (0.38)	< 0.001
**Number of desaturations**^a^	< 1 *N = 25*	Area < 80% SpO_2_ limit	0.01 (0.004)	0.01 (0.003)	0.54
		No of desaturations	0.39 (0.05)	0.52 (0.07)	< 0.001
	1–3 *N = 28*	Area < 80% SpO_2_ limit	0.07 (0.02)	0.03 (0.01)	0.08
		No of desaturations	1.41 (0.16)	1.48 (0.17)	0.21
	3–6 *N = 24*	Area < 80% SpO_2_ limit	0.26 (0.09)	0.18 (0.06)	0.41
		No of desaturations	3.58 (0.39)	2.85 (0.31)	< 0.001
	> 6 *N = 35*	Area < 80% SpO_2_ limit	0.46 (0.13)	0.20 (0.06)	0.04
		No of desaturations	7.5 (0.66)	4.26 (0.38)	< 0.001
**Gestational age**	< 26 weeks *N = 50*	Area < 80% SpO_2_ limit	0.19 (0.07)	0.11 (0.04)	0.08
		No of desaturations	3.92 (0.86)	2.59 (0.57)	< 0.001
	26–28 weeks *N = 51*	Area < 80% SpO_2_ limit	0.16 (0.05)	0.12 (0.04)	0.32
		No of desaturations	3.92 (0.71)	2.97 (0.54)	< 0.001
	≥ 28 weeks *N = 11*	Area < 80% SpO_2_ limit	0.05 (0.03)	0.01 (0.005)	0.17
		No of desaturations	1.03 (0.39)	0.23 (0.09)	0.009
**Hematocrit**	< 25% *N = 34*	Area < 80% SpO_2_ limit	0.15 (0.06)	0.08 (0.03)	0.12
		No of desaturations	3.53 (0.72)	2.08 (0.42)	< 0.001
	25–30% *N = 61*	Area < 80% SpO_2_ limit	0.12 (0.04)	0.07 (0.02)	0.08
		No of desaturations	2.83 (0.54)	2.21 (0.42)	< 0.001
	≥ 30% *N = 17*	Area < 80% SpO_2_ limit	0.19 (0.07)	0.11 (0.04)	0.26
		No of desaturations	3.47 (1.01)	2.23 (0.65)	0.001
**Respiratory support**	Invasive *N = 74*	Area < 80% SpO_2_ limit No of desaturations	0.13 (0.04) 2.12 (0.37)	0.07 (0.02) 1.48 (0.26)	0.03 < 0.001
	Non-invasive *N = 37*	Area < 80% SpO_2_ limit No of desaturations	0.15 (0.06) 4.47 (0.92)	0.09 (0.04) 3.01 (0.62)	0.19 < 0.001

Data before and after red blood cell (RBC) transfusion are expressed as estimated means(SE). The area under the 80% SpO_2_ curve is presented as mean %/second and the number of desaturations as mean number/hour. For both parameters, the mean is calculated over the 24 h period before and after transfusion

^*a*^Mean number of desaturations per hour in the 24 h before transfusion

**Fig. 4:**
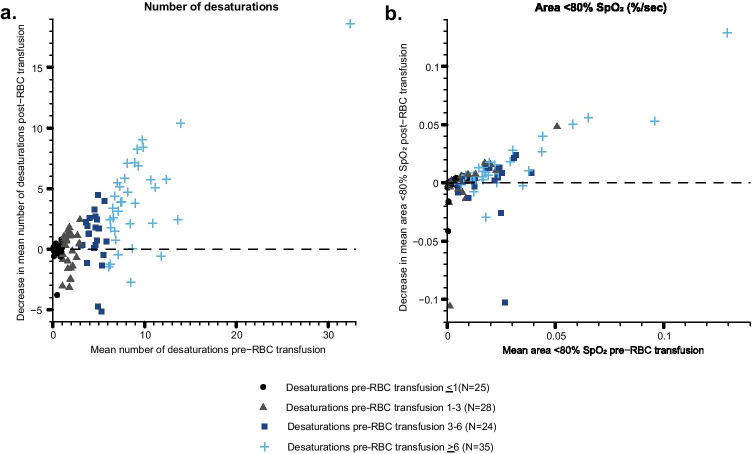
Decrease in **a** the mean number of desaturations and **b** area under the 80% oxygen saturation (SpO_2_) limit in the 24 h after red blood cell (RBC) transfusion in relation to the mean values in the 24 h before transfusion. Data are presented individually for all RBC transfusions, marked in the desaturation subgroups

On an individual level, the number of desaturations decreased in 12/25 (48.0%) of RBC transfusions with desaturations ≤ 1, in 16/28 (57.1%) of transfusions with desaturations 1–3, in 19/24 (79.2%) of transfusions with desaturation 3–6, and in 30/35 (85.7%) of transfusions with desaturations ≥ 6 (Fig. 4a). The area under the 80% SpO_2_ limit decreased in 13/25 (52.0%) of RBC transfusions with desaturations ≤ 1, in 18/28 (64.3%) of transfusions with desaturations 1–3, in 17/24 (70.8%) of transfusions with desaturation 3–6, and in 28/35 (80.0%) of transfusions with desaturations ≥ 6 (Fig. 4b). The higher the mean number of desaturations in the 24 h prior to RBC transfusion, the higher the decrease in the number of desaturations after RBC transfusion (Fig. 4a).

### Respiratory parameters

**Fig. 5:**
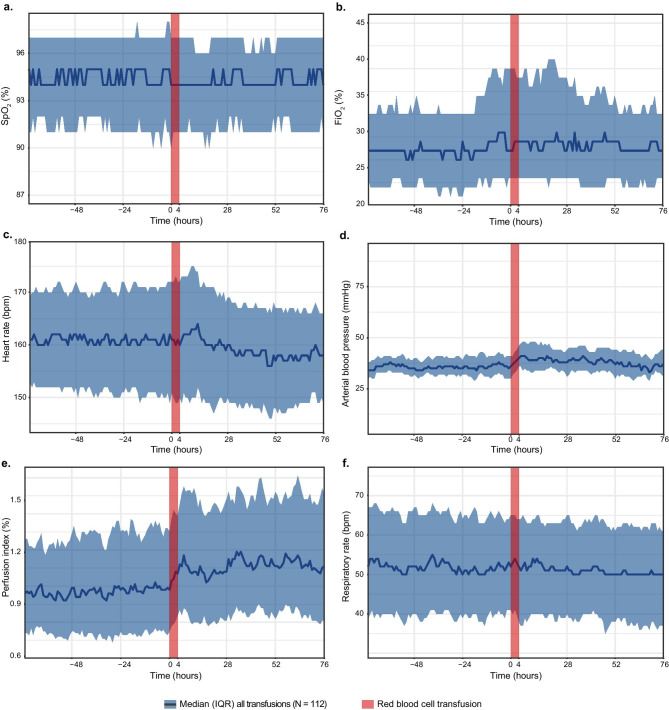
Graphs with data of all red blood cell transfusions displayed over 3 days before until 3 days after transfusion; **a** oxygen saturation (SpO_2_), **b** fraction of inspired oxygen (FiO_2_), **c** heart rate (bpm), **d** mean arterial blood pressure (mmHg), **e** perfusion index (%), **f** respiratory rate (bpm). Data are presented as median [interquartile range (IQR)] because of the skewed distribution

In the overall group, the SpO_2_ in the 24 h before RBC transfusion differed not significantly from that in the 24 h after RBC transfusion (*p* = 0.97) (Fig. 5a). The FiO_2_ slightly increased before RBC transfusion and decreased after transfusion, although not statistically significant (*p* = 0.07) (Fig. 5b). Overall, the respiratory rate was not significantly influenced by RBC transfusion (*p* = 0.29) (Fig. 5f).

### Circulatory parameters

The median heart rate increased in the first hours after RBC transfusion and then decreased below the median heart rate pre-transfusion (Fig. 5c). However, the estimated mean heart rate in the 24 h before and after transfusion did not differ significantly (*p* = 0.89). The mean perfusion index increased from 1.05 (0.05) to 1.18 (0.05) after RBC transfusion in all RBC transfusions (*p* < 0.001) (Fig. 5e). The mean arterial blood pressure increased from 28.5 (3.9) to 33.5 (3.4) mmHg after RBC transfusion, although not statistically significant (*p* = 0.09) (Fig. 5d).

## Discussion

This study gives insight in the effects of RBC transfusions on the number of desaturations and the area under the 80% SpO_2_ limit derived from respiratory monitor data. Both the number of desaturations and area under the 80% SpO_2_ limit, and consequently the hypoxic burden, decreased after RBC transfusion. The higher the number of desaturations before a transfusion, the larger the effect. The number of desaturations decreased after transfusion, irrespective of the respiratory support type, Ht values, or GA at birth. For the area under the 80% SpO_2_ limit, this effect was only observed in invasively ventilated infants. In those infants already had no desaturations prior to RBC transfusion, no beneficial effect could be objectified.

RBC transfusion guidelines are mostly based on Hb level cutoffs, and thresholds for transfusion vary largely amongst national guidelines and local NICUs [3]. Symptomatic anemia, however, does not occur at a predefined Hb level [28]. In our study, RBC transfusion had a positive effect on the number of desaturations, especially in those experiencing the most desaturations before transfusion. Similarly, a decrease in intermittent hypoxia and apneas after RBC transfusion was found in previous studies [6, 9, 29]. However, none of these studies stratified for the frequency of desaturations before transfusion. The decrease in the number of desaturations and the area under the 80% SpO_2_ after transfusion was found to be inconsistent in this study. Although the number of desaturations decreased significantly in almost all subgroups, the area under the 80% SpO_2_ limit only decreased significantly in infants with the highest number of desaturations and invasive ventilation. This might indicate that the decrease in the occurrence of desaturations is possibly a decrease in the least severe desaturations.

Hypoxemic episodes are known to be associated with late death and disability in preterm infants [26]. A post hoc analysis of the premature infants in need of transfusion (PINT) trial suggested a cognitive deficit at 18–21 months in infants who were held at a lower hemoglobin level [30]. A recent systematic review also suggested that anemia and RBC transfusions have impact on the developing brain of preterm infants, possibly by the effect on cerebral oxygenation [31]. Causality of impaired neurodevelopment with hypoxemic episodes was, however, not confirmed by two recent RCTs comparing restrictive to liberal transfusion strategy [18, 19]. These trials provided clinically relevant and meaningful information regarding the neurodevelopmental outcomes with different transfusion thresholds in large cohorts of preterm infants. The duration of respiratory support and age at last use of caffeine therapy as a measure of hypoxia were equal in both treatment groups. Other more subtle manifestations of hypoxia, such as apnea, were not reported as this was not the main focus of the studies. Most importantly, the level of hypoxia before and the decrease after RBC transfusion were therefore also not reported and could differ between infants within a treatment group. Although speculative, RBC transfusion at a certain level of desaturations could decrease the hypoxic burden, irrespective of the degree of anemia, and consequently might prevent hypoxia-associated long-term adverse outcomes in preterm infants.

RBC transfusion, on the other hand, is not without risk. Although no advantageous outcomes of RBC transfusion are found on long-term mortality and severe morbidity [18, 19, 32–35], it is unknown whether more subtle long-term adverse effects of RBC transfusions itself occur in preterm infants. However, a previous study found negative associations with platelet transfusions, and we could argue that a blood transfusion leads to an immunologic and inflammatory response [36].

A reported inverse correlation between Ht values and the probability of future apneas suggests that the effect of RBC transfusion on apneas is mediated by increased oxygen transport capacity [29]. Apneas can be of central, obstructive, or mixed origin, which cannot be distinguished from the used respiratory data. RBC transfusion is likely not effective in case of obstructive apneas, and, although speculative, this could explain why hypoxia is not always reduced completely after transfusion in our study. Additionally, apneas and the related hypoxia in preterm infants are not specific for anemia only; these could be due to other neonatal diseases as well, such as pulmonary disease or sepsis.

Two-thirds of our study population were invasively ventilated during transfusion and were consequentially less likely to have desaturations. Also, the severity and etiology might differ from desaturations during non-invasive ventilation. In about a quarter of the transfusions during invasive respiratory support, no desaturations occurred before transfusion. When desaturations were present, the frequency decreased significantly after transfusion, which suggests that central apneas with desaturations might still occur during invasive ventilation. A previous study found improved oxygenation after RBC transfusion in mechanically ventilated preterm infants, although data on oxygenation was only collected at three time points [37]. For invasively ventilated infants, the number of desaturations only might not be sufficient to indicate the need for transfusion. Ht or Hb levels need to be evaluated before deciding on the need for RBC transfusion.

In our study population, the FiO_2_ was slightly increased during the day before transfusion, probably as a result of manual adjustments as response to an increase in desaturations. Clinicians might already have noticed the impaired oxygenation. The available data did not indicate alternations in the circulatory parameters before transfusion, suggesting that circulatory parameters do not indicate the need for transfusion. After RBC transfusion, median arterial blood pressure and pulse index increased immediately as a result of increased blood volume. Heart rate slightly increased in the first hours after transfusion, likely due to volume load, followed by a decrease below the average heart rate pre-transfusion.

A limitation of this study is that co-interventions such as respiratory support and caffeine therapy could have been intensified during transfusion to improve an infant’s respiratory status. In 11 cases, invasive ventilation was started within 12 h before transfusion, and in 16 cases, an additional caffeine loading dose was administered in the 24 h prior to transfusion. In this study, the effect of RBC transfusion on the respiratory status was adjusted for these co-interventions in the mixed effects models. Additionally, we could not assess whether the presence of clinical signs, such as a pale skin, contributed to the decision to give a RBC transfusion, as clinical signs were likely underreported.

A more individualized approach would be key to optimize the risk benefit ratio of medical interventions such as RBC transfusions. The large availability of physiological monitor data enabled us to objectively detect desaturations and hypoxia associated with anemia and to evaluate alterations in a patient’s clinical status. Algorithms detecting relevant patterns in physiological monitor data could provide more individualized care and help predict which patients are most likely to benefit. Unnecessary transfusions, with the risk for adverse effects, could then be avoided.

In conclusion, RBC transfusions could help decrease oxygen desaturations and the hypoxic burden in preterm newborns, especially when desaturations are more frequent prior to transfusion. Transfusion thresholds alone do not seem to indicate who will benefit from RBC transfusion. In future care, algorithms to detect relevant patterns in bedside cardiorespiratory monitor data can be used to predict which preterm infant will benefit from RBC transfusion.

## Supplementary information

Below is the link to the electronic supplementary material.

Supplementary file1 (EPS 1632 KB)

## Data Availability

The data that support the findings of this study are available from the corresponding author upon reasonable request.

## Abbreviations

FiO_2_: Fraction of inspired oxygen
GA: Gestational age
Hb: Hemoglobin
Ht: Hematocrit
IQR: Interquartile range
MAP: Mean airway pressure
NICU: Neonatal intensive care unit
RBC: Red blood cell
SE: Standard error
SpO_2_: Oxygen saturation
